# Proline-rich tyrosine kinase 2 via enhancing signal transducer and activator of transcription 3-dependent cJun expression mediates retinal neovascularization

**DOI:** 10.1038/srep26480

**Published:** 2016-05-23

**Authors:** Raj Kumar, Nikhlesh K. Singh, Gadiparthi N. Rao

**Affiliations:** 1 Department of Physiology, University of Tennessee Health Science Center, Memphis, TN 38163, USA

## Abstract

Despite the involvement of proline-rich tyrosine kinase 2 (Pyk2) in endothelial cell angiogenic responses, its role in pathological retinal angiogenesis is not known. In the present study, we show that vascular endothelial growth factor A (VEGFA) induces Pyk2 activation in mediating human retinal microvascular endothelial cell (HRMVEC) migration, sprouting and tube formation. Downstream to Pyk2, VEGFA induced signal transducer and activator of transcription 3 (STAT3) activation and cJun expression in the modulation of HRMVEC migration, sprouting and tube formation. Consistent with these observations, hypoxia induced activation of Pyk2-STAT3-cJun signaling axis and siRNA-mediated downregulation of Pyk2, STAT3 or cJun levels substantially inhibited hypoxia-induced retinal endothelial cell proliferation, tip cell formation and neovascularization. Together, these observations suggest that activation of Pyk2-mediated STAT3-cJun signaling is required for VEGFA-induced HRMVEC migration, sprouting and tube formation *in vitro* and hypoxia-induced retinal endothelial cell proliferation, tip cell formation and neovascularization *in vivo*.

Retinal revascularization is one of the major causes of vision loss in Western countries^1^. Vision loss in retinopathy of prematurity, proliferative diabetic retinopathy and age related macular degeneration is the result of aberrant angiogenesis caused by the involvement of many growth factors^2^,^3^. Angiogenesis is a complex process which involves interaction between multiple cell types and various signal transduction mechanisms^3^. Impairment in any of these coordinated events leads to aberrant vessel formation^3^,^4^. Among various growth factors identified thus far, vascular endothelial growth factor A (VEGFA) is the most potent cytokine that mediates ischemia-induced neovascularization in ocular pathologies^5^,^6^. VEGFA exerts its effects via activating various signaling events, including tyrosine phosphorylation of its receptors, and their downstream effectors^7^. However, the mechanisms underlying VEGFA-induced retinal pathological angiogenesis are not completely understood.

Proline-rich tyrosine kinase 2 (Pyk2) is a non-receptor tyrosine kinase, which is involved in calcium-mediated ion channel regulation and mitogen-activated protein kinase (MAPK) activation^8^. Previous studies have shown that focal adhesion kinase (FAK) and Pyk2 share structural properties with overlapping cellular functions^9^. In this context, it should be noted that Pyk2 compensates endothelial-specific lack of FAK in the modulation of angiogenesis^9^. Although Pyk2 has been shown to mediate integrin and vascular endothelial-cadherin-mediated cell adhesion and migration, very little is known about its role in retinal endothelial cell sprouting and pathological neovascularization^10^,^11^,^12^,^13^.

Signal transducer and activator of transcription 3 (STAT3) is a transcription factor that enhances the expression of various genes involved in many cellular processes such as cell survival and cell proliferation^14^. STAT3 is activated by both tyrosine and serine phosphorylation^15^,^16^. Tyrosine phosphorylation of STAT3 results in its dimerization and translocation to the nucleus where it enhances transcription of genes^14^. On the other hand, serine phosphorylation of STAT3 results in its transcriptional activation through enhanced recruitment of other transcriptional cofactors^16^. STAT3 has been implicated to mediate mitogenic, angiogenic and anti-apoptotic effects of growth factors and cytokines in endothelial cells^14^,^17^. Furthermore, STAT3 has been reported to be involved in the regulation of VEGF expression, tumor angiogenesis and metastasis^18^. It has also been shown that binding of hypoxia-inducible factor (HIF)-1α and STAT3 to VEGFA promoter is necessary for hypoxia-induced VEGFA expression^19^. In contrary to a large number of studies pointing a role for STAT3 in angiogenesis^18^,^19^, some reports have shown that STAT3 inhibits VEGFA-induced angiogenic events^20^. Given the presence of STAT3 in the retina and its involvement in tumor angiogenesis, we examined its role in retinal neovascularization.

In the present study, we report that Pyk2 via STAT3-cJun signaling mediates VEGFA-induced HRMVEC migration, sprouting and tube formation *in vitro* and hypoxia-induced retinal endothelial cell proliferation, tip cell formation and neovascularization *in vivo*.

## Results

### Pyk2 mediates VEGFA-induced HRMVEC migration, sprouting and tube formation

**Figure 1:**
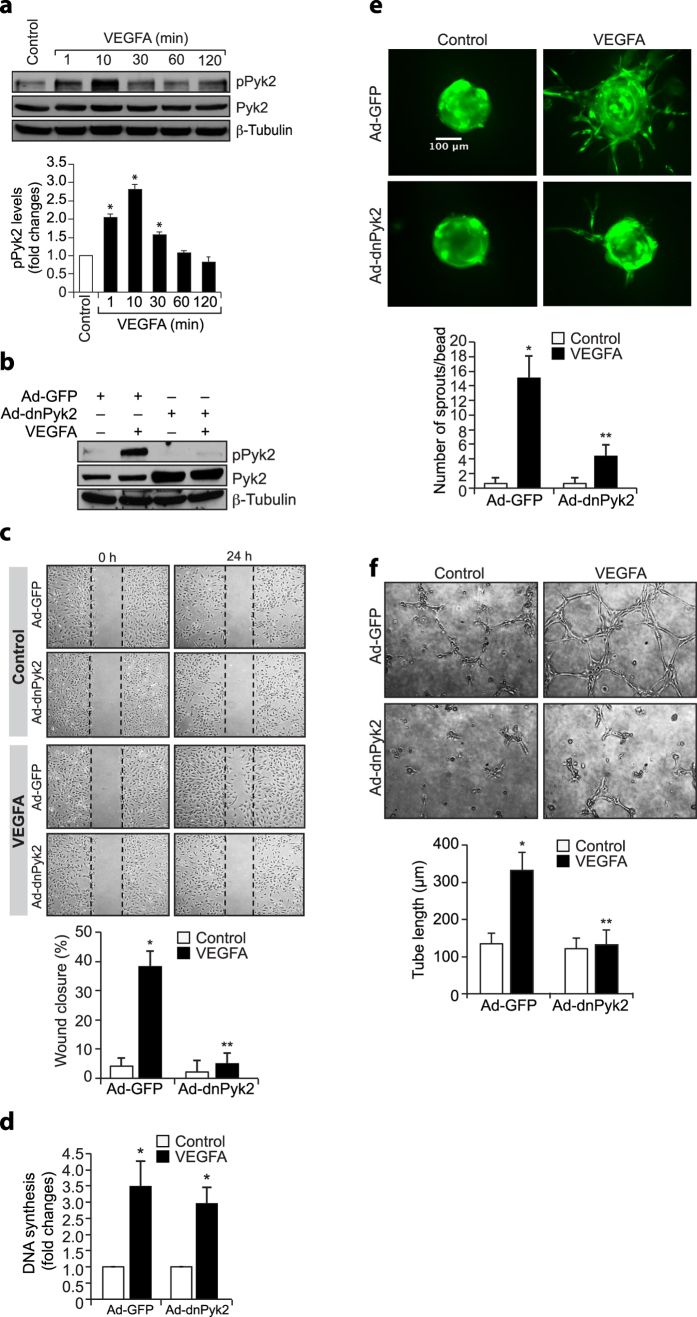
Pyk2 regulates VEGF-induced angiogenic events in HRMVECs. (**a**) Quiescent HRMVECs were treated with and without VEGF (40 ng/ml) for the indicated periods and cell extracts were prepared. Equal amounts of protein from control and each treatment were analyzed by Western blotting for pPyk2 levels using its specific antibodies. The blot was reprobed for total Pyk2 or β-tubulin for normalization. (**b**) HRMVECs were transduced with Ad-GFP or Ad-dnPyk2 (40 moi), growth-arrested, treated with and without VEGF (40 ng/ml) for 10 min, cell extracts were prepared and equal amounts of protein from control and each treatment were analyzed for pPyk2 levels and the blot was reprobed for total Pyk2 or β-tubulin to show the over expression of dnPyk2 or normalization. (**c–f**) All the conditions were the same as in panel **b** except that after growth-arrest, cells were subjected to VEGFA-induced migration (**c**), DNA synthesis (**d**), sprouting (**e**), or tube formation (**f**) assays. The bar graphs represent quantitative analysis of three independent experiments. The values are presented as Mean ± SD. *p < 0.01 vs control or Ad-GFP; **p < 0.01 vs Ad-GFP ± VEGFA.

Both developmental and pathological angiogenesis are mostly governed by VEGFA^21^. Various non receptor tyrosine kinases such as Janus kinase 2 (Jak2) and Pyk2 are involved in VEGFA-induced endothelial cell migration and proliferation^22^,^23^. In order to understand the role of these non-receptor tyrosine kinases in VEGFA-induced angiogenic events, we have studied the time course effect of VEGFA on Pyk2 tyrosine phosphorylation in HRMVECs. VEGFA induced Pyk2 tyrosine phosphorylation in a time-dependent manner with maximum effect at 10 min (Fig. 1a). Furthermore, dominant negative mutant-mediated blockade of Pyk2 activation without affecting DNA synthesis attenuated VEGFA-induced HRMVEC migration, sprouting and tube formation (Fig. 1b–f). These results indicate that activation of Pyk2 is required for VEGFA-induced HRMVEC migration, sprouting and tube formation but not proliferation.

### Hypoxia-induced retinal neovascularization requires Pyk2 activation

**Figure 2:**
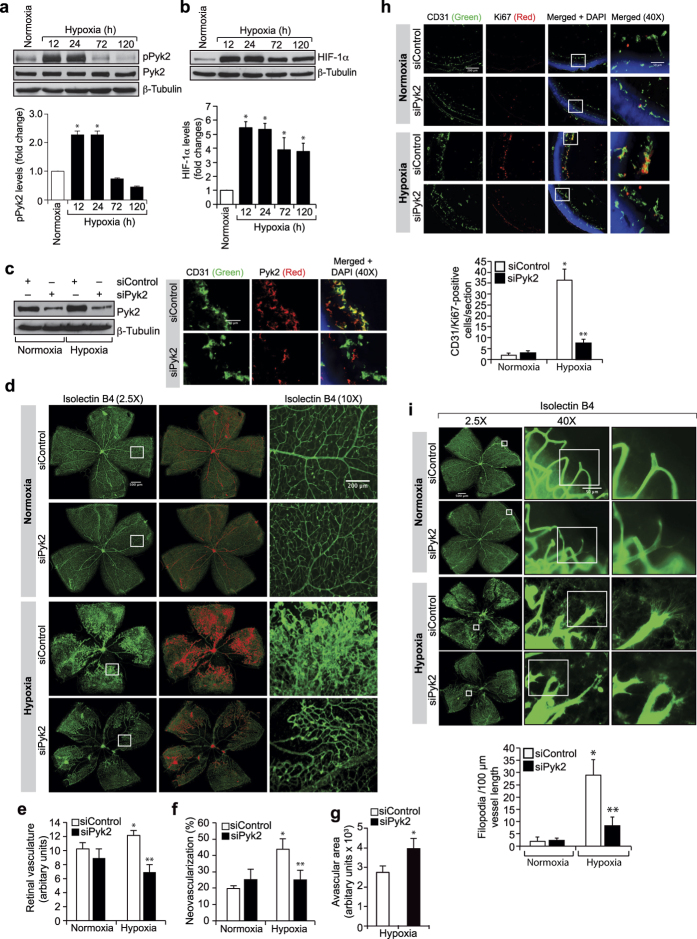
Pyk2 mediates hypoxia-induced retinal neovascularization. (**a,b**) Eyes from normoxic or various time periods of hypoxic pups were enucleated, retinas isolated and retinal extracts were analyzed for pPyk2 or HIF-1α levels and normalized to total Pyk2 or β-tubulin. (**c**) All the conditions were same as in panel **a** except that the pups were received intravitreal injections of the indicated siRNA at P12, P13, and P15. At P17 eyes were enucleated and either retinas isolated and analyzed for Pyk2 and β-tubulin levels to show the effect of the siRNA on its target and off target molecules, respectively, or fixed, cross-sections made and stained for CD31 and Pyk2. (**d**) All the conditions were the same as in panel **c** except that the retinas were isolated, fixed, stained with isolectin B4, flat mounts made and examined for retinal neovascularization. Retinal vascularization is shown in the first column. Neovascularization is highlighted in red in the second column. The third column shows the selected rectangular areas of the images in the first column under 10x magnification. (**e–g**) Retinal vasculature (**e**), neovascularization (**f**) and avascular area (**g**) were determined as described in “Materials and Methods.” (**h**) All the conditions were same as in panel **c** except that pups were injected with control or Pyk2 siRNA intravitreally only at P12 and P13. At P15 eyes were enucleated, fixed, cross-sections made and stained for CD31 and Ki67. The right column shows the higher magnification (40X) of the areas selected by rectangular boxes in the left column images. (**i**) All the conditions were the same as in panel **c** except that at P17 the enucleated eyes were fixed, retinas isolated, stained with isolectin B4, flat mounts made and examined for EC filopodia formation. The first column shows the whole flat mounts and the second and third columns show the selected rectangular areas of the images in the first column under microscopic (40X) and digital magnifications, respectively. The bar graphs represent quantitative analysis of 6 retinas. The values were presented as Mean ± SD. *p < 0.01 vs normoxia; **p < 0.01 vs siControl ± hypoxia.

To emphasize the significance of the *in vitro* findings *in vivo*, we next studied the role of Pyk2 in retinal neovascularization using a mouse model of oxygen-induced retinopathy (OIR). Mice pups were kept at 75% oxygen from P7 to P12 and at P12 they were returned to room air to develop the relative hypoxia. Retinal extracts were prepared at various time periods of hypoxia (starting from P12) and analyzed by Western blotting for Pyk2 tyrosine phosphorylation. Hypoxia induced tyrosine phosphorylation of Pyk2 in a time-dependent manner with maximum effect at 12 hrs (Fig. 2a). To validate hypoxic condition, we also measured HIF-1α levels. HIF-1α was induced by several-fold in response to hypoxia with a maximum effect at 12 hrs (Fig. 2b). To test the role of Pyk2 in retinal neovascularization, Pyk2 levels were depleted by its siRNA molecules and tested for its effects in hypoxia-induced retinal neovascularization. Downregulation of Pyk2 levels profoundly reduced hypoxia-induced retinal neovascularization as observed by a decrease in endothelial cell proliferation, a decrease in the number of tufts and filopodia and disappearance of blood vessel dilatation with increased avascular area (Fig. 2c–i). These findings indicate that Pyk2 activation is required for hypoxia-induced retinal EC proliferation, tip cell formation and neovascularization.

### STAT3 activation is required for VEGFA-induced HRMVEC migration, sprouting and tube formation

**Figure 3:**
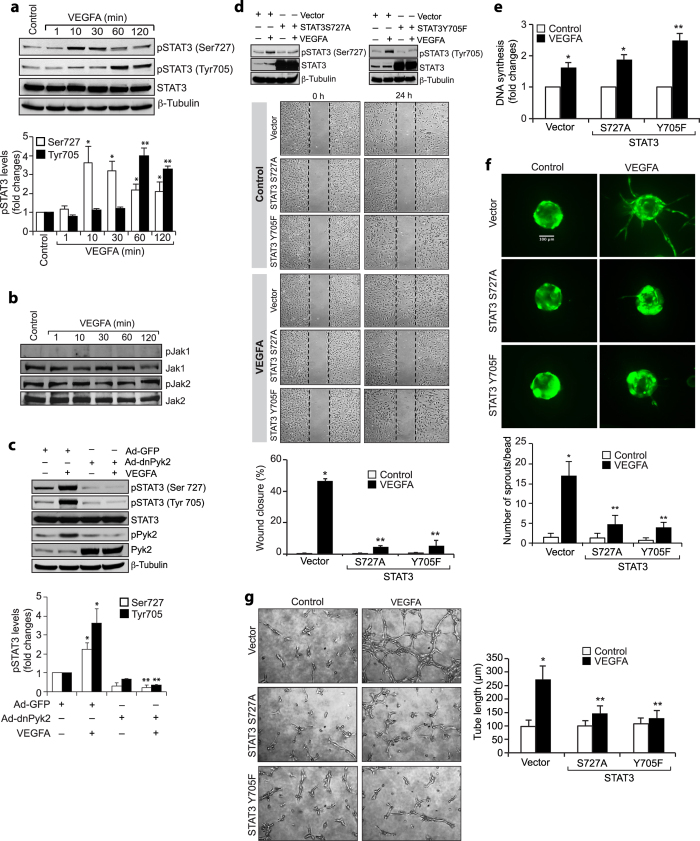
Pyk2 mediates STAT3 activation in VEGF-induced HRMVEC angiogenic responses. (**a,b**) An equal amount of protein from control and various time periods of VEGFA (40 ng/ml)-treated HRMVECs was analyzed by Western blotting for pSTAT3, pJak1 and pJak2 levels and the blots were reprobed for total STAT3, Jak1, Jak2 or β-tubulin for normalization. (**c**) Cells were transduced with Ad-GFP or Ad-dnPyk2 (40 moi), growth-arrested, treated with and without VEGFA (40 ng/ml) for 10 min or 60 min, cell extracts were prepared and analyzed by Western blotting for pSTAT3 and pPyk2 levels and the blots were reprobed for total STAT3, Pyk2 or β-tubulin to show the over expression of dnPyk2 or normalization. (**d**) Upper panel: Cells were transfected with empty vector or mutant STAT3 (STAT3S727A or STAT3Y705F), growth-arrested, treated with and without VEGFA (40 ng/ml) for 10 min or 60 min, cell extracts were prepared and analyzed by Western blotting for pSTAT3 levels and the blots were reprobed for total STAT3 or β-tubulin to show the over expression of mutant STAT3 or normalization. Lower panel: All the conditions were the same as in upper panel except that after growth-arrest cells were subjected to VEGFA-induced cell migration assay. (**e–g**) All the conditions were the same as in panel **d** except that after growth-arrest cells were subjected to VEGFA-induced DNA synthesis (**e**), sprouting (**f**) or tube formation (**g**) assays. The bar graphs represent quantitative analysis of three independent experiments. The values are presented as Mean ± SD. *p < 0.01 vs control or vector or Ad-GFP; **p < 0.01 vs vector ± VEGFA or Ad-GFP ± VEGFA.

**Figure 4:**
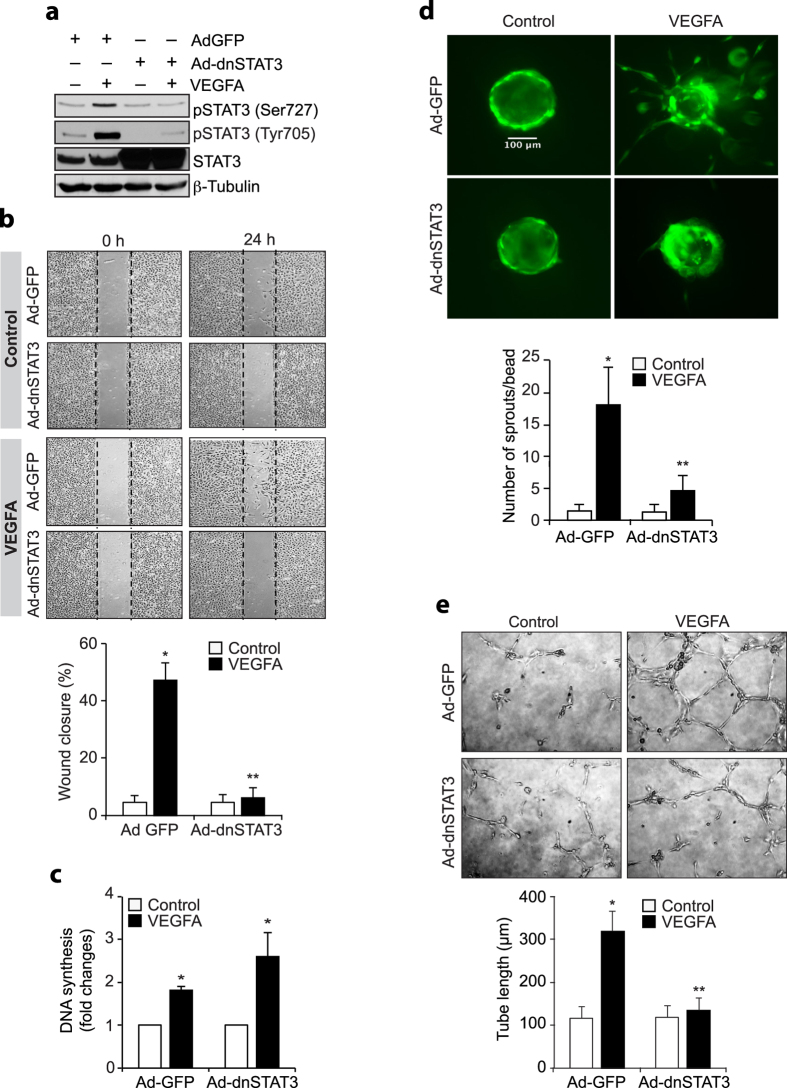
STAT3 mediates VEGF-induced HRMVEC migration, sprouting and tube formation. (**a**) Cells were transduced with Ad-GFP or Ad-dnSTAT3, growth-arrested, treated with and without VEGFA (40 ng/ml) for 10 min or 60 min, cell extracts were prepared and analyzed by Western blotting for pSTAT3 levels and the blots were reprobed for total STAT3 or β-tubulin to show the over expression of dnSTAT3 or normalization. (**b**–**e**) All the conditions were the same as in panel **a** except that after growth-arrest cells were subjected to VEGFA-induced migration (**b**), DNA synthesis (**c**), sprouting (**d**) or tube formation (**e**) assays. The bar graphs represent quantitative analysis of three independent experiments. The values are presented as Mean ± SD. *p < 0.01 vs Ad-GFP; **p < 0.01 vs Ad-GFP ± VEGFA.

Since STAT3 plays a role in VEGFA-induced tumor angiogenesis^24^, and Pyk2 mediates epidermal growth factor-induced STAT3 activation^25^, we explored the potential role of Pyk2 in VEGFA-induced STAT3 activation in HRMVECs. VEGFA induced both serine and tyrosine phosphorylation of STAT3 in HRMVECs (Fig. 3a). Since Janus kinases (JAKs) mediate STAT3 activation in tumor angiogenesis, we first tested the effect of VEGFA on Jak1 and Jak2 tyrosine phosphorylation in HRMVECs. VEGFA had little or no effect on Jak1 or Jak2 tyrosine phosphorylation, suggesting lack of a role for these tyrosine kinases in STAT3 activation (Fig. 3b). We next tested the role of Pyk2 in VEGFA-induced STAT3 activation. Interestingly, dominant negative mutant-mediated blockade of Pyk2 activation inhibited VEGFA-induced STAT3 phosphorylation at both serine and tyrosine residues in HRMVECs (Fig. 3c). This observation indicates that Pyk2 mediates VEGFA-induced STAT3 activation. To understand the functional importance of STAT3 activation in VEGFA-induced angiogenic responses in HRMVECs, we studied the effects of its serine (S727A) and tyrosine (Y705F) mutants in VEGF-induced HRMVEC migration, proliferation, sprouting and tube formation. Interference with STAT3 activation by forced expression of its S727A or Y705F mutant without affecting DNA synthesis abolished VEGFA-induced HRMVEC migration, sprouting and tube formation (Fig. 3d–g). To confirm these findings, we also tested the effect of its double mutant (Y705F/S727A) in VEGFA-induced HRMVEC angiogenic responses. Blockade of STAT3 phosphorylation both at serine and tyrosine residues simultaneously without affecting DNA synthesis inhibited VEGFA-induced HRMVEC migration, sprouting and tube formation (Fig. 4a–e).

### Pyk2-mediated STAT3 activation is required for hypoxia-induced retinal neovascularization

**Figure 5:**
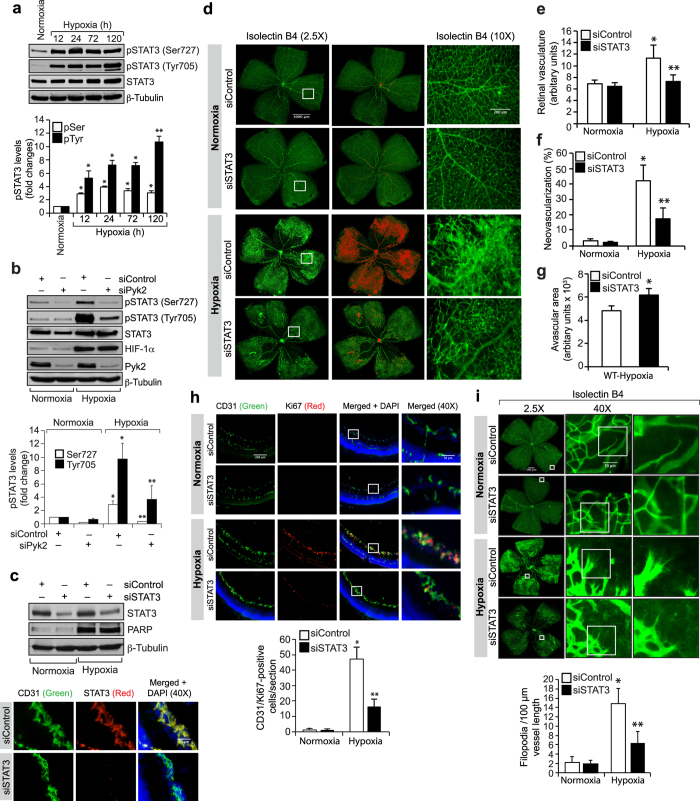
STAT3 mediates retinal neovascularization. (**a**) Retinal extracts of normoxic and hypoxic pups were analyzed for pSTAT3 levels and normalized for total STAT3 or β-tubulin. (**b**) Mice pups were given intravitreal injections of the indicated siRNA at P10, and P11 and at P13 the retinal extracts were prepared and analyzed by Western blotting for the indicated molecules. (**c,d**) All the conditions were the same as in panel **b** except that pups were injected with control or STAT3 siRNA intravitreally at P12, P13 and P15 and at P17 the retinal extracts were analyzed for STAT3, PARP and β-tubulin levels to show the efficacy of the siRNA on its target and off target molecules or the enucleated eyes were fixed, cross-sections made and stained for CD31 and STAT3 or the retinas were isolated, fixed, stained with isolectin B4, flat mounts were made and examined for retinal neovascularization. Retinal vascularization is shown in the first column. Neovascularization is highlighted in red in the second column. The third column shows the selected rectangular areas of the images in the first column under 10x magnification. (**e–g**) Retinal vasculature (**e**), neovascularization (**f**) and avascular area (**g**) were determined as described in “Materials and Methods.” (**h**) All the conditions were the same as in panel **c** except that pups were injected with control or STAT3 siRNA intravitreally at P12 and P13 and at P15 the enucleated eyes were fixed, cross-sections made and stained for CD31 and Ki67. The right column shows the higher magnification of the selected areas in the left column images. (**i**) Conditions were the same as in panel **c** except that at P17 the eyes were enucleated, fixed, retinas isolated, stained with isolectin B4, flat mounts made and examined for EC filopodia formation. The first column shows the whole flat mounts and the second and third columns show the microscopic and digital magnifications of the selected rectangular areas in the first column. The bar graphs represent quantitative analysis of 6 retinas. The values were presented as Mean ± SD. *p < 0.01 vs normoxia; **p < 0.01 vs siControl + hypoxia.

In order to understand the role of STAT3 in retinal neovascularization, we first tested its activation by OIR. Hypoxia induced both serine and tyrosine phosphorylation of STAT3 (Fig. 5a). To understand the mechanisms of STAT3 activation in hypoxic retina, we studied the role of Pyk2. Depletion of Pyk2 levels by its siRNA molecules without affecting HIF-1α levels reduced hypoxia-induced STAT3 phosphorylation both at serine and tyrosine residues suggesting that Pyk2 mediates STAT3 activation in hypoxic retina as well (Fig. 5b). To understand the role of STAT3 in retinal neovascularization, we used siRNA approach. Downregulation of STAT3 levels using its siRNA molecules significantly reduced retinal neovascularization with increased avascular area compared to mice injected with control siRNA (Fig. 5c–g). Depletion of STAT3 levels also attenuated retinal EC proliferation as well as tip cell formation (Fig. 5h,i). To rule out the possibility that the decreased retinal EC proliferation by STAT3 depletion was due to increased apoptosis, we measured cleaved poly (ADP-ribose) polymerase (PARP) levels, a marker of apoptosis^26^. Although hypoxia induced cleaved PARP levels, no significant differences were found in its levels between control and STAT3-depleted retinas (Fig. 5c). These observations confirm that the decreased retinal EC proliferation by STAT3 downregulation was not due to increased EC apoptosis. These results indicate that STAT3 activation downstream to Pyk2 is required for retinal neovascularization.

### Pyk2-STAT3 signaling enhances cJun expression in the mediation of retinal neovascularization

**Figure 6:**
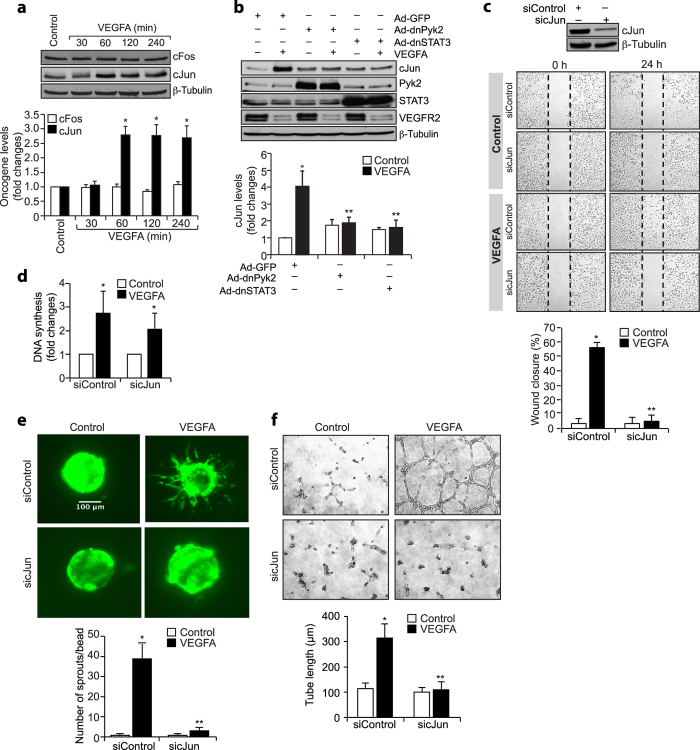
VEGF-induced HRMVEC angiogenic responses require cJun expression. (**a**) Growth-arrested HRMVECs were treated with and without VEGFA (40 ng/ml) for the indicated time periods, cell extracts were prepared and analyzed by Western blotting for cFos and cJun levels. (**b**) Cells were transduced with Ad-GFP, Ad-dnPyk2, or Ad-dnSTAT3, growth-arrested, treated with and without VEGFA (40 ng/ml) for 60 min, cell extracts were prepared and an equal amount of protein from control and each treatment was analyzed by Western blotting for cJun and VEGFR2 levels. The blots in panels **a** and **b** were reprobed for Pyk2, STAT3 or β-tubulin to show the over expression of dnPyk2 or dnSTAT3 or normalization. (**c**) Upper panel: HRMVECs were transfected with control or cJun siRNA and 2 days later, cell extracts were prepared and analyzed for cJun and β-tubulin levels to show the efficacy of the siRNA on its target and off target molecules. Lower panel: All the conditions were the same as in upper panel except that after transfection cells were subjected to VEGFA-induced migration assay. (**d–f**) All the conditions were the same as in lower panel **c** except that cells were subjected to VEGFA-induced DNA synthesis (**d**), sprouting (**e**), or tube formation (**f**) assays. The bar graphs represent quantitative analysis of three independent experiments. The values are presented as Mean ± SD. *p < 0.01 vs siControl; **p < 0.01 vs siControl + VEGFA.

STAT3 mediates the growth promoting effects of angiogenic factors such as VEGFA. In addition, many reports showed that cJun/cFos interact with STAT3 in the transcriptional induction of several genes. To understand whether VEGFA-induced STAT3 activation has any role in cJun or cFos expression, we tested the time course effect of VEGFA on the expression of these proto-oncogenes. VEGFA induced cJun but not cFos expression in a time-dependent manner in HRMVECs (Fig. 6a). Next, we tested the role of Pyk2-STAT3 signaling in VEGFA-induced cJun expression. Dominant negative mutant-mediated inhibition of either Pyk2 or STAT3 activation blocked VEGFA-induced cJun expression (Fig. 6b). As expected, upon treatment with VEGFA, VEGFR2 levels were found to be downregulated, perhaps, due to its phopshorylation and internalization^27^. However, blockade of Pyk2-STAT3 signaling had no effect on VEGFR2 levels (Fig. 6b). To understand the functional aspects of cJun in VEGFA-induced angiogenic responses, we tested its role in VEGFA-induced HRMVEC migration, sprouting, and tube formation. Suppression of cJun levels by its siRNA molecules attenuated VEGFA-induced HRMVEC migration, sprouting, and tube formation (Fig. 6c–f).

**Figure 7:**
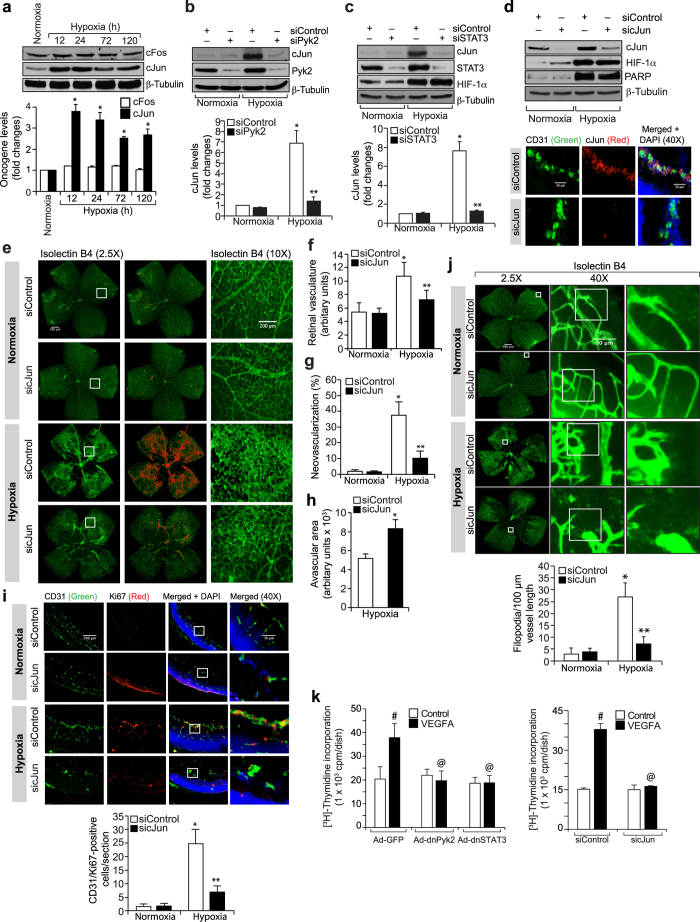
Retinal neovascularization requires cJun expression. (**a**) The normoxic and hypoxic retinal extracts were analyzed for cFos and cJun levels and normalized to β-tubulin. (**b,c**) Mice pups were injected with the indicated siRNA intravitreally at P10, and P11 and at P13 the retinas were analyzed for cJun levels and the blots were reprobed for the indicated molecules to show the efficacy and specificity of the siRNA. (**d,e**) Mice pups were injected with the indicated siRNA intravitreally at P12, P13, and P15 and at P17 retinas were analyzed for cJun, HIF-1α and β-tubulin levels or the enucleated eyes were fixed, cross-sections made and stained for CD31 and cJun or retinas isolated, stained with isolectin B4, flat mounts made and examined for neovascularization. Retinal vascularization is shown in the first column. Neovascularization is highlighted in red in the second column. The third column shows the selected areas of the images in the first column under 10x magnification. (**f–h**) Retinal vasculature (**f**), neovascularization (**g**) and avascular area (**h**) were shown. (**i**) Pups were injected with the indicated siRNA intravitreally at P12 and P13 and at P15 eyes were fixed, cross-sections made and stained for CD31 and Ki67. The right column shows the higher magnification of the selected areas in the left column images. (**j**) Pups were injected with the indicated siRNA intravitreally at P12, P13 and P15 and at P17 eyes were enucleated, fixed, retinas isolated, stained with isolectin B4, flat mounts made and examined for EC filopodia formation. The first column shows the whole flat mounts and the second and third columns show the microscopic and digital magnifications of the selected areas in the first column images. (**k**) MRMVECs that were transduced with the indicated adenovirus or transfected with the indicated siRNA and growth-arrested were subjected to VEGFA-induced DNA synthesis. The bar graphs represent quantitative analysis of 6 retinas. The values were presented as Mean ± SD. *p < 0.01 vs normoxia; **p < 0.01 vs siControl + hypoxia; ^#^*p* < 0.01 vs Ad-GFP or siControl; ^@^*p* < 0.01 vs Ad-GFP + VEGFA or siControl + VEGFA.

To understand the role of cJun in hypoxia-induced retinal neovascularization, we have first studied the effect of hypoxia on its expression. Without affecting cFos levels, hypoxia induced cJun expression in the retina in a time-dependent manner (Fig. 7a). Furthermore, downregulation of Pyk2 or STAT3 levels by their siRNA molecules inhibited hypoxia-induced cJun expression (Fig. 7b,c). Downregulation of STAT3 levels had no effect in hypoxia-induced HIF-1α expression (Fig. 7c). Since cJun regulates ischemia-induced angiogenesis^28^, we next explored its role in hypoxia-induced retinal neovascularization. Depletion of cJun levels by its siRNA molecules while having no effect on HIF-1α expression, blunted hypoxia-induced retinal EC proliferation and filopodia formation leading to a reduction in retinal neovascularization with increased avascular area (Fig. 7d–j). Since inhibition of Pyk2-STAT3-cJun signaling does not affect VEGFA-induced HRMVEC proliferation but blocks hypoxia-induced retinal EC proliferation in mice, we asked the question whether these differential effects of Pyk2, STAT3 and cJun in the modulation of HRMVEC versus mouse retinal EC proliferation were due to species variations. To address this postulation, we have studied the role of Pyk2-STAT3-cJun signaling in VEGFA-induced mouse retinal microvascular endothelial cell (MRMVEC) proliferation. Dominant negative mutant-mediated Pyk2 or STAT3 activation or siRNA-mediated downregulation of cJun levels substantially inhibited VEGFA-induced DNA synthesis in MRMVECs (Fig. 7k).

## Discussion

Retinal neovascularization is a common manifestation of various retinal disorders, including retinopathy of prematurity (ROP), proliferative diabetic retinopathy (DR) and age related macular degeneration^2^,^3^,^29^. All these ocular pathologies are associated with increased expression of angiogenic factors that stimulate neovascularization in the retina^30^,^31^. Most of these diseases are characterized by increased endothelial cell proliferation, vascular permeability and inflammation^2^,^3^,^4^. VEGFA is a pro-angiogenic cytokine that mediates all these processes and is the primary factor involved in neovascularization^32^. VEGFA signaling in retinal neovascularization is complex and involves various signal transduction pathways^7^,^12^,^23^. In the present study, we show that Pyk2-STAT3-dependent cJun expression plays a role in VEGFA-induced HRMVEC migration, sprouting and tube formation and hypoxia-induced retinal endothelial cell proliferation, tip cell formation and neovascularization. Since interference with activation of Pyk2, STAT3 or cJun had no effect on hypoxia-induced HIF-1α levels, it is likely that Pyk2-STAT3-cJun signaling plays a role downstream to, or independent of HIF-1α in hypoxia-induced retinal neovascularization. These findings provide the first conclusive evidence on the role of Pyk2-STAT3-cJun signaling in pathological retinal angiogenesis.

Pyk2 is a focal adhesion tyrosine kinase, which regulates multiple signaling events required for focal adhesion turnover and migration of cells^33^. Pyk2 knockout mice have exhibited defective endothelial cell migration and tubulogenesis following hind-limb ischemia^10^. The role of Pyk2 in endothelial cell sprouting has also been reported^34^,^35^. In addition, we have shown that Pyk2 is involved in endothelial tight junction disruption^36^. In the present study, we present evidence that Pyk2 mediates VEGFA-induced HRMVEC migration, sprouting and tubulogenesis and hypoxia-induced retinal endothelial cell proliferation, tip cell formation and neovascularization. However, it was noted that downregulation of Pyk2 levels only inhibited hypoxia-induced EC proliferation in the mouse retina, but not HRMVEC proliferation. These differential effects of Pyk2 in mouse versus human retinal microvascular endothelial cell proliferation may be attributed to species differences as clarified later in the “Discussion.” Pyk2 has been shown to mediate epidermal growth factor-induced STAT3 activation in HeLa cells^37^. In the present study, we demonstrate that Pyk2 mediates both VEGFA and hypoxia-induced STAT3 serine and tyrosine phosphorylation. Many studies have shown that STAT3 plays a role in angiogenesis, tumor development and metastasis in response to various stimulants^38^,^39^,^40^. It has also been demonstrated that STAT3 is activated by hypoxia in microglia, astrocytes, and neurons^41^,^42^. Using rat 50/10 oxygen-induced retinopathy model^43^, previous studies have shown that neovascular retinal vessels exhibit increased STAT3 activity^44^. Using HRMVECs and mouse OIR model, our present observations demonstrate that depletion of STAT3 levels significantly reduces endothelial cell migration, sprouting and tube formation *in vitro* and retinal endothelial cell proliferation, tip cell formation and neovascularization *in vivo*. Thus, while a large body of data indicates a role for STAT3 in angiogenesis^38^,^39^,^40^, some studies have reported that STAT3 activation blocks retinal vascularization^45^. It should be noted that if STAT3 was involved in vessel regression then one would expect that depletion of STAT3 levels results in enhanced vessel growth in response to hypoxia, which was not the case in the present study. Therefore, it is likely that STAT3 activation is required for enhanced neovascularization rather than vessel regression.

Upon activation, STAT3 translocates form the cytoplasm to the nucleus and increases the expression of its target genes involved in cell proliferation, migration, invasion or angiogenesis depending on the stimulus^46^,^47^,^48^. It has also been reported that cJun interacts with STAT3 and enhances IL-6 response element transactivation^49^. Besides these observations, our findings demonstrate that VEGFA induces cJun expression downstream to Pyk2-STAT3 signaling in the regulation of HRMVEC migration, sprouting and tube formation. A role for cJun in 15(*S*)-hydroxyeicosatetraenoic acid and epoxyeicosatrienoic acid-induced angiogenesis has also been reported^26^,^50^. Corroborating these *in vitro* findings, cJun expression in hypoxic retina was dependent on both Pyk2 and STAT3 activation and its downregulation suppresses hypoxia-induced endothelial cell proliferation, tip cell formation and retinal neovascularization. Since inhibition of Pyk2-STAT3-cJun signaling only blocks VEGFA-induced MRMVEC but not HRMVEC proliferation, it should be pointed out that this signaling modulates EC growth in a species-specific manner. Many studies have shown that AP-1 plays a role in the regulation of expression of matrix metalloproteinases (MMPs) involved in cell migration and invasion^51^,^52^,^53^. Since cJun via homodimerization or heterodimerization with the members of the Fos family of proto-oncogene proteins can form an active AP-1 complex^54^, it is possible that cJun might be involved in the regulation of expression of MMPs in mediating VEGFA and hypoxia-mediated EC angiogenic responses. The reports that Pyk2 and STAT3 modulate the expression of MMPs^55^,^56^ also support this view as Pyk2-STAT3 signaling axis is required for both VEGFA and hypoxia-induced cJun expression.

**Figure 8:**
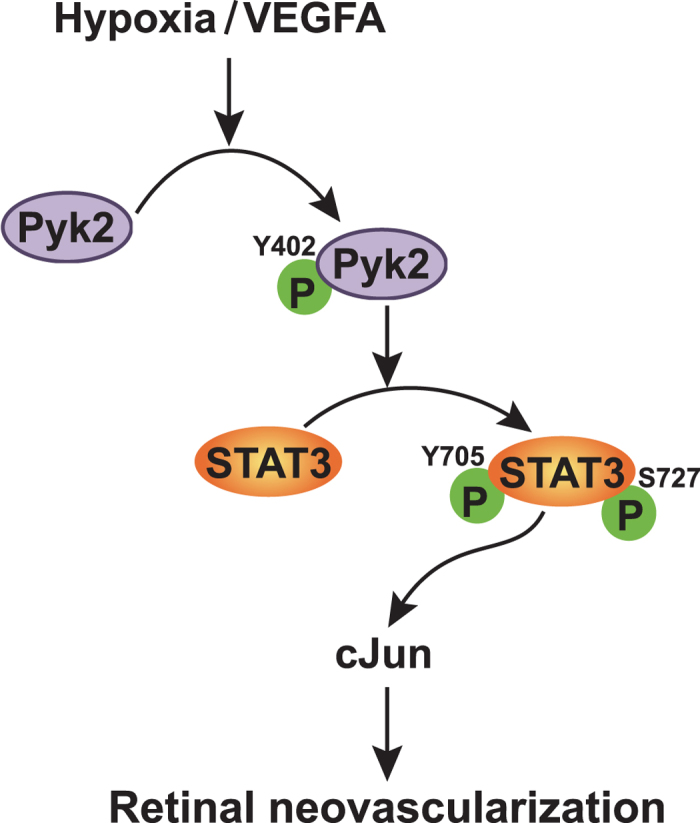
Schematic diagram showing the potential signaling of Pyk2 in retinal neovascularization.

In summary, as depicted in Fig. 8, the present study demonstrates the importance of Pyk2-STAT3-dependent cJun expression in mediating retinal endothelial cell migration, proliferation, sprouting and neovascularization.

## Material and Methods

### Reagents

Growth factor-reduced Matrigel (354230) was obtained from BD Biosciences (Bedford, MA). Recombinant human VEGF165 (293-VE-010/CF) was bought from R&D Systems (Minneapolis, MN). Anti-β-tubulin (SC-9104), anti-c Jun (SC-1694), anti-Jak1 (Sc-295) and anti-STAT3 (SC-482) antibodies were purchased from Santa Cruz Biotechnology (Santa Cruz, CA). Anti-PARP (9542), anti-pPyk2 (3291), anti-pSTAT3 Ser727 (9134), anti-pSTAT3 Tyr705 (9131) and anti-VEGFR2 (2479) were obtained from Cell Signaling Technology (Beverly, MA). Anti-pJak2 (07-606) and anti-Jak2 antibodies were purchased from Millipore (Billerica, MA). Anti-CD31 (550274) antibody was bought from BD Pharmingen (Palo Alto, CA). Rat anti-mouse CD31 antibody (550330) and nylon mesh (352340) were purchased from BD Biosciences (San Jose, CA). Anti-HIF-1α (ab113642), anti-Ki67 (ab15580) and anti-Pyk2 (ab32571) antibodies were obtained from Abcam (Cambridge, MA). Collagenase type I (LS004196) was bought from Worthington (Lakewood, NJ). Control nontargeting small interfering RNA (siRNA) (D-001810-10), mouse cJun siRNA (ON-TARGET plus SMARTpool L-043776-00-0010), human-cJun siRNA (ON-TARGET plus SMARTpool L-003268-00-005) and mouse STAT3 siRNA (ON-TARGET plus SMARTpool L-040794-01) were purchased from Dharmacon (Pittsburgh PA,). Mouse Pyk2 siRNA (MSS208196) and sheep anti-rat Dynabeads (11035) were obtained from Life Technologies Corporation (Grand Island, NY). Rhodamine-phalloidin (00027) was purchased from Biotium, Inc. (Hayward, CA). Alexa Fluor 488-conjugated goat anti-rat IgG, Alexa Fluor 568-conjugated goat anti-rabbit IgG, Hoechst 33342, isolectin B4-594, and Prolong Gold antifade reagent were bought from Molecular Probes (Eugene, OR). [^3^H]-Thymidine (S. A. 20 Ci/mM) was obtained from Perkin Elmer (Boston, MA). STAT3 S727A pRc/CMV (Addgene Plasmid #8708) and STAT3 Y705F Flag pRc/CMV (Addgene plasmid #8709) were gifts from James E. Darnell, Jr.^14^.

### Adenoviral vectors

The construction of Ad-GFP, Ad-dnPyk2, and Ad-dnSTAT3 was described previously^40^,^57^,^58^.

### Animals

C57BL/6 pregnant mice at E16 were obtained from Charles River Laboratories, Wilmington, MA and bred at University of Tennessee Health Science Center’s vivarium. All the experiments involving animals were approved by the Animal Care and Use Committee of the University of Tennessee Health Science Center, Memphis, TN. The methods were carried out according to approved Institutional guidelines.

### Cell culture

Human retinal microvascular endothelial cells (HRMVECs) were purchased from Applied Cell Biology Research Institute (Kirkland, WA) and cultured in medium 131 containing microvascular growth supplements (MVGS), 10 μg/ml gentamycin, and 0.25 μg/ml amphotericin B. Cultures were maintained at 37 °C in a humidified 95% air and 5% CO_2_ atmosphere. HRMVECs with passage numbers between 5 and 10 were synchronized in medium 131 without MVGS for 24 hrs and used to perform the experiments unless otherwise indicated.

### Mouse retinal microvascular endothelial cell isolation

Eyes from 3 wk-old C57BL/6 mice pups were enucleated, retinas dissected out, minced, and digested in 4 ml of collagenase type I (1 mg/ml in serum-free DMEM) for 60 min at 37 °C. Following digestion, equal volume of DMEM containing 10% FBS was added and the cellular digest was filtered through a sterile 40 μm nylon mesh and centrifuged at 1000 rpm for 5 min to pellet the cells. The cell pellet was washed twice with DMEM containing 10% FBS, resuspended in 1.0 ml of DMEM with 10% FBS and incubated with sheep anti-rat IgG magnetic beads pre-coated with rat anti-mouse CD31 antibody [the beads were washed three times with serum-free DMEM and incubated with rat anti-mouse CD31 antibody for overnight at 4 °C (10 μl antibody/50 μl beads in DMEM)]. After affinity binding, magnetic beads were washed six times with DMEM containing 10% FBS and the bound cells were plated onto a single well of a 24-well plate. Mouse retinal endothelial cells were cultured in EGM-2 medium containing 20% FBS at 37 °C in a humidified 95% air and 5% CO_2_ and growth-arrested in EBM-2 medium without any supplements for 24 hrs to perform the experiments.

### Cell migration

HRMVEC migration was measured by wound-healing assay. Briefly, endothelial cells were plated in ibidi culture insert at 2 × 10^4^ cells/chamber, grown to full confluence and growth-attested in medium 131 without any growth supplements for 24 hrs. After the growth-arresting period, the ibidi inserts were removed and 1 ml of medium 131 containing 5 mM hydroxyurea was added. Cells were treated with and without VEGFA (40 ng/ml) for 24 hrs and the migrated cells were observed under Nikon Eclipse TS100 microscope with 4X/0.13 magnification and the images were captured with a Nikon Digital Sight DS-L1 camera. The cell migration was calculated using ImageJ and expressed as percent wound closure (total area at 0 hrs-area not occupied by the cells at 24 hrs/total area at 0 hrs X 100).

### DNA synthesis

DNA synthesis was measured by [^3^H]-thymidine incorporation^59^. HRMVECs were plated onto 6-well plates, allowed to grow to 70–80% confluence, growth-arrested for 24 hrs, treated with and without VEGFA (40 ng/ml) for 30 hrs and pulse-labeled with 1 μCl/ml of [^3^H]-thymidine for the last 24 hrs of the 30-hrs incubation period. Cells were collected by trypsinization followed by centrifugation and the cell pellet was resuspended in cold 10% (w/v) trichloroacetic acid. After vortexing the mixture was kept on ice for 30 min and was then passed through a GF/F glass microfiber filter. The filter was washed once with cold 5% tricholoroacetic acid, once with cold 70% ethanol, dried, placed in a scintillation vial containing the scintillation cocktail and the radioactivity was measured in liquid scintillation counter (Beckman LS 3801). DNA synthesis was expressed as counts/min/dish.

### Tube formation

Three hundred microliters of HRMVEC suspension at a concentration of 5 × 10^5^ cells/ml were added to each well of a twenty four-well culture plate that was pre-coated with 280 μl of growth factor-reduced Matrigel. Cell were treated with vehicle or VEGFA (40 ng/ml) for 6 hrs at 37 °C. Tube formation was observed under an inverted microscope (Eclipse TS100, Nikon, Tokyo, Japan) and the images were captured using a CCD color camera (KP-D20AU: Hitachi, Ibaraki, Japan) and Apple iMovie 7.1.4 software. The tube length was calculated using NIH ImageJ and expressed in micrometers.

### Western blotting

Cell or tissue extracts containing an equal amount of protein were resolved by electrophoresis on 0.1% SDS and 10% polyacrylamide gels. The proteins were transferred to a nitrocellulose membrane and after blocking in either 5% nonfat dry milk or BSA, the membrane was probed with the appropriate primary antibodies followed by incubation with horseradish-peroxidase-conjugated secondary antibodies. The antigen-antibody complexes were detected using an enhanced chemiluminescence detection reagent kit (Amersham Biosciences).

### Transfection

HRMVECs at 70% confluence in medium 131 lacking MVGS were transfected with control or target siRNA at a final concentration of 100 nM using Lipofectamine 2000 transfection reagent following the manufacturer’s instructions. Six hours after transfection, medium was replaced with fresh medium 131 containing MVGS and antibiotics for 24 hrs and growth-arrested in medium 131 without any supplements for 24 hrs before using for experimentation.

### Transduction

HRMVECs were transduced with control or target adenovirus at a final concentration of 40 moi in medium 131 containing MVGS and antibiotics for overnight. Medium was replaced with fresh medium 131 containing both MVGS and antibiotics and after 24 hrs of incubation cells were growth-arrested overnight in medium 131 without any supplements and used for experimentation.

### Sprouting

Three-dimensional sprouting was performed as described previously^59^. Briefly, the transduced/transfected HRMVECs were coated onto cytodex beads overnight and embedded in fibrin (2 mg/ml fibrinogen +0.625 units/ml of thrombin) gels. Fibroblasts were plated on top of the fibrin gels and incubated at 37 °C for the indicated time periods. The beads were examined on day 3 and day 5 for sprouting under Ziess inverted microscope (Observer.Z1: original magnification X10/NA 0.45) and the fluorescence images were captured by Ziess AxioCam MRm camera using microscope operating and image analysis software AxioVision 4.7.2 (Carl Ziess Imaging Solutions GmbH). The number of sprouts on each bead were counted using NIH imageJ. Sprouting was expressed as number of sprouts/bead.

### Oxygen-induced retinopathy (OIR)

OIR was performed and quantified as described by Connor *et al.*^60^. C57BL/6 mice pups along with dams were exposed to 75% oxygen from P7 to P12 and at P12 they returned to room air to develop the relative hypoxia. Mice pups of the same age kept at room air were used as controls. At P17 mice pups were sacrificed, eyes were enucleated and fixed in 4% (w/v) paraformaldehyde for 1 hr at room temperature. Retinas were isolated, stained with isolectin B4, flat mounts were made, coverslips were placed and examined under a Zeiss inverted fluorescence microscope (Zeiss Observer. Z1). Retinal vasculature was quantified by calculating the ratio of fluorescence intensity to the total retinal area. Retinal neovascularization was measured by first setting a scale with a tolerance point of 50 pixels based on the fluorescence intensity in the screenshot using Nikon NIS-Elements software version AR 3.1. Neovascularity (values above 50 pixels tolerance set point) was highlighted in red and then quantified by dividing the fluorescence intensity in the highlighted area by the total fluorescence intensity in the screenshot (n = 6 eyes).

### Intravitreal injections

During the indicated time periods of hyperoxia or hypoxia, pups were administered with control, or the targeted siRNA at 1 μg/0.5 μl/eye) by intravitreal injections using a 33G needle.

### Immunofluorescence staining

After hyperoxia, mouse pups were returned to room air for 3 days, after which time they were sacrificed, eyes enucleated, fixed in optimal cutting temperature compound, and cryosections were prepared. To identify proliferating ECs, after blocking in normal goat serum, the cryosections were probed with rabbit anti-mouse Ki67 antibodies (1:100) and rat anti-mouse CD31 antibodies (1:100) followed by incubation with Alexa Fluor 568-conjugated goat anti-rabbit and Alexa Fluor 488-conjugated goat anti-rat secondary antibodies. The sections were observed under Zeiss inverted microscope (Observer.Z1: original magnification X40/NA 0.6 or original magnification X10/NA 0.45) and the fluorescence images were captured by AxioCam MRm camera using the microscope operating and image analysis software AxioVision 4.7.2 (Carl Zeiss Imaging Solutions GmbH). The retinal EC proliferation was quantified by counting Ki67- and CD31-positive cells that extended anterior to the inner limiting membrane per section (n = 6 eyes, 3 sections/eye).

### Statistics

All experiments were repeated 3 times and the data are presented as Mean ± Standard Deviation (SD). The treatment effects were analyzed by one-way ANOVA followed by Tukey’s post-hoc comparisons and *p* values < 0.05 were considered statistically significant.

## Additional Information

**How to cite this article**: Kumar, R. *et al.* Proline-rich tyrosine kinase 2 via enhancing signal transducer and activator of transcription 3-dependent cJun expression mediates retinal neovascularization. *Sci. Rep.* **6**, 26480; doi: 10.1038/srep26480 (2016).
